# Salivary Melatonin in Relation to Depressive Symptom Severity in Young Adults

**DOI:** 10.1371/journal.pone.0152814

**Published:** 2016-04-04

**Authors:** Isak Sundberg, Mia Ramklint, Mats Stridsberg, Fotios C. Papadopoulos, Lisa Ekselius, Janet L. Cunningham

**Affiliations:** 1 Department of Neuroscience, Psychiatry, Uppsala University, Uppsala, Sweden; 2 Department of Medical Sciences, Biochemical Endocrinology, Uppsala University, Uppsala, Sweden; Benito Menni Complejo Asistencial en Salud Mental, SPAIN

## Abstract

Reduced levels of melatonin have been associated with severe depression. The aim was to investigate the correlation between salivary melatonin and dimensional measures of depressive symptom severity in young adult psychiatric patients. Levels of melatonin were analyzed in six saliva samples during waking hours from 119 young adult patients under outpatient psychiatric care. Melatonin levels were tested for association with the severity of depressive symptoms using the self-rating version of the Montgomery Åsberg Depression Rating Scale (MADRS-S). Where possible, depressive symptoms were assessed again after 6±2 months of treatment. Response was defined as decrease in MADRS-S by ≥50% between baseline and follow-up. Patients with levels of melatonin in the lowest quartile at bedtime had an increased probability of a high MADRS-S score compared to those with the highest levels of melatonin (odds ratio 1.39, 95% CI 1.15–1.69, p<0.01). A post hoc regression analysis found that bedtime melatonin levels predicted response (odds ratio 4.4, 95% CI 1.06–18.43, p<0.05). A negative relationship between salivary melatonin and dimensional measures of depressive symptom severity was found in young patients under outpatient psychiatric care. Bedtime salivary melatonin levels may have prognostic implications.

## Introduction

Melatonin is produced in the pineal gland and regulates sleep. It is secreted in a circadian manner, with a peak during the evening and night. A replicated finding is lower peripheral nighttime and/or 24-hour melatonin levels in depressed patients compared to healthy controls [1–8]. The studies showing melatonin reduction in depression have largely been conducted on inpatients with more severe depression. Other studies, in outpatient settings [9, 10] or with mixed patient groups [11], have not found significant differences in melatonin levels between patients with depression and controls. There are even reports of higher melatonin concentrations in depressed individuals as compared to healthy controls [12]. A negative correlation between depression severity and 24-hour amplitude of plasma melatonin [5], as well as a positive correlation to degree of circadian misalignment, have been found [13]. A negative correlation between evening melatonin levels and the degree of depressed mood and reality disturbance has also been reported [14]. Other publications have found no significant relationship between levels of melatonin and indices of depression severity [9, 12, 14–16]. The majority of studies are on small samples with a limited range of depression severity, and confounding factors are not always accounted for in the analysis. Factors such as age [17, 18], gender [19], use of oral contraceptives [20], antidepressant medication [21], BMI [22, 23], beta blockers [24], and season [25] influence melatonin secretion.

New medications for depression that target melatonin receptors have reached the clinic. Dysregulation of melatonin secretion may be useful as a clinical biomarker in order to identify a subgroup of patients, with potential implications for prognosis and treatment choice. New methods for measurement of melatonin in saliva allow for easy multiple sampling by the patient at home. Salivary melatonin has been shown to significantly correlate with blood levels of melatonin [26].

The aim of this study was to investigate the relationship between salivary melatonin levels and the severity of depressive symptoms. The cohort consists of young adults in a general psychiatry outpatient clinical setting with a wide range of depressive symptoms. Symptoms were assessed using the Montgomery Åsberg Depression Rating Scale—Self-Assessment (MADRS-S) score as a dimensional measure of depression symptom severity [27–29]. Based on the literature, we hypothesized a negative relationship between evening melatonin and dimensional measures of depressive symptoms.

## Materials and Methods

### Ethics and patient consent

The study was approved by the Regional Ethics Committee in Uppsala (Dnr. 2012/81, Dnr. 2012/81/1, and Dnr. 2013/219). All participants gave written consent.

### Patients and clinical data and controls

“Uppsala Psychiatry Patient samples” (UPP) is an infrastructure for the collection of biological material from patients with psychiatric symptoms at the Department of General Psychiatry at Uppsala University Hospital, Sweden. Blood, saliva, and feces samples are collected, together with information from the case record and questionnaire-based demographic and symptomatic data. This initiative was started in October 2012 and is ongoing. Data for the current study was gathered within the framework for UPP during the time period October 2012 to May 2014. Consecutive new patients at an outpatient clinic for young adults between the ages of 18 and 25 that met the criteria for any psychiatric diagnosis according to the Diagnostic and Statistical Manual of Mental Disorders, 4th Edition (DSM-IV) were included in this study. Initial structured diagnostics included an interview by a doctor or fully trained psychologist using either the Structured Clinical Interview for DSM-IV Axis I Disorders-Clinical Version (SCID-1) [30] or Mini-International Neuropsychiatric Interview (MINI) [31, 32]. Within 24 hours of saliva and blood collection, depressive symptoms were assessed using the MADRS-S. This time point is referred to as “baseline” in the text. Information was also gathered regarding sociodemographic data, medical history, and treatment. The Sheehan Disability Scale (SDS) was used to rate disability [33]. The SDS is a self-report tool where the study subjects rate the extent of disability, ranging from 0 (not at all) to 10 (extremely) to which school/work, social life, and home/family life is impaired by his or her symptoms. The sum of these scores gives a global impairment rating ranging from 0 to 30, with higher scores indicating greater functional impairment. The Swedish version of the SDS was used with permission from D. V. Sheehan. When available in the medical journal, MADRS-S scale assessment at clinical follow-up after 4 to 8 months and closest to 6 months after baseline was documented. A research assistant examined participants regarding height and weight. Body mass index was calculated (kg/m^2^).

Out of 722 consecutive patients, 300 agreed to participate in the Uppsala Psychiatric Patient samples (UPP) study. Of these, 125 completed saliva sampling. Six patients were omitted because they did not fulfill criteria for any DSM-IV Axis 1 diagnosis. A total of 119 patients with a DSM-IV Axis 1 disorder completed saliva sampling and were enrolled in this study. In the population (n = 119), MADRS-S total scores ranged between 4 and 46, with a median value of 23.

Those who completed saliva sampling (n = 119) differed from the total group (n = 300) with regards to gender (85% vs. 75% female, p = 0.029) and BMI (24.4 vs. 21.5, p = 0.01). However, total MADRS-S score (23.3 vs. 21.5) and the distribution of diagnoses; depressive episode (57% vs. 50%) and any anxiety disorder (66% vs. 64%) were not statistically different. See Table 1 for participant characteristics.

**Table 1:** Participant characteristics.

	All patients	No Current Depressive episode	Current Depressive episode
	n (%)	n (%)	n (%)
N (%)	119 (100)	51 (42.9)	68 (57.1)
Female	102 (85.7)	43 (84.3)	59 (86.8)
Male	17 (14.3)	8 (9)	9 (13.2)
Age (mean)	21	21	21
BMI (mean)	23.3	23.6	24.6
MADRS-S	23.6	18.4	27.5
**Diagnosis**			
Unipolar disorder, single episode	39 (32.8)	10 (19.6)	29 (42.6)
Unipolar disorder, recurrent	41 (34.5)	18 (35.3)	23 (33.8)
Bipolar disorder, type 1	4 (3.4)	2 (3.9)	2 (2.9)
Bipolar disorder, type 2 and unspecified	25 (21.0)	11 (21.7)	14 (20.9)
Any anxiety disorder	79 (66.4)	31 (60.8)	49 (72.0)
Medication*			
SSRI/SNRI**	60 (50.4)	25 (49.0)	35 (51.5)
Antipsychotics	6 (5.0)	4 (7.8)	2 (2.9)
Mood stabilizers	8 (6.7)	7 (13.7)	1 (1.5)
Oral Anticonception	30 (25.2)	17 (33.3)	13 (19.1)

* Treatment at the time of saliva sampling

** SSRI/SNRI: Selective serotonin re-uptake inhibitors/ Serotonin–norepinephrine re-uptake inhibitors

### Saliva collection and analysis

Participants were instructed to collect saliva samples at six time points during the waking hours of one day: when waking up; 30 minutes after waking up but before breakfast; at 11.00 hours; 30 minutes after lunch; at 22.00 hours, and just before going to bed. Time points were chosen to capture melatonin onset before sleep and, based on new knowledge that the gastrointestinal tract secretes melatonin, salivary melatonin variation during waking hours and after meals. Participants were carefully instructed and received written guidance on the method of sample collection. Saliva was collected using inert polymer cylindrical swabs (10 mm x 30 mm), which were then placed in a storage tube that consists of a large outer tube with a small insert and snap cap (swabs and tubes from Salimetrics Europe Ltd. Suffolk. UK) and kept in the refrigerator until delivery to the lab within 48 hours. Participants were instructed not to eat or drink 30 minutes before sampling. The participants documented collection times. To ensure compliance, the research assistant verified collection times and sampling method with the patient upon receipt, and samples not collected as instructed were excluded. Of maximally 714 (6x119) hormone measurements, 35 (4.9%) were missing from 17 patients due to mistakes in saliva sampling or insufficient saliva volume. Total assay variability was <11%.

Upon receipt, tubes were centrifuged and stored at -20°C until analysis. Salivary melatonin was measured with competitive ELISA (Direct Salivary Melatonin Elisa EK-DSM. Bühlmann Laboratories AG.Schönenbuch. Switzerland). Analyses were performed at the routine laboratory of the Department of Clinical Chemistry at Uppsala University Hospital, Uppsala, Sweden. The laboratory is certified by a Swedish government authority (Swedac).

### Blood sample collection and analysis for exploratory analysis

Given melatonin’s newly described role in glucose regulation and well-known function as an antioxidant, glycated hemoglobin (HbA1C) as a measure of long-time glucose levels and oxidized low-density lipoprotein (oxidized-LDL) as a measure of oxidative stress were analyzed in a subgroup of the population. Blood samples were collected from patients in 7 ml vials containing EDTA on the same day they completed baseline questionnaires. Plasma from one vial and whole blood from the other were aliquoted and stored at -80°C and only thawed once at the time of analysis. EDTA-plasma was analyzed for oxidized-LDL using an Elisa method (Catalog number 10-1143-01. Mercodia. Uppsala. Sweden). Total coefficient of variation was <9%. Whole EDTA blood was analyzed for HbA1C by the Department of Clinical Chemistry at Uppsala University Hospital using the automated instrument Cobas c501. Reagents were purchased from Roche Diagnostics (Reagent Cobas Integra^®^ Hba1c Gen2 D/S. art.nr. 04528123190. Roche Diagnostics. Bromma. Sweden) and analysis was conducted according to manufacturer’s instructions. Total coefficient of variation was <4%.

### Statistics

All statistical analyses were conducted with the Statistical Package for the Social Sciences (SPSS) Statistics Version 22. Patients enrolled in this study were compared to patients in UPP who did not provide saliva (internal drop-outs) regarding gender, BMI, MADRS-S score, and diagnosis of current depressive episode and anxiety disorder using the Mann Whitney test and independent samples median test.

### Normality distribution and data transformation

Prior to all analyses, the continuous variables were screened for normality distribution with the Shapiro Wilks test of normality, P > 0.05, and visually estimated normal distribution (histogram. Q-Q- plot. Box-plot). The total MADRS-S scores were normally distributed. BMI was not normally distributed. Salivary melatonin was not normally distributed. Non-parametric tests were performed when analyzing non-normally distributed variables. Where values for melatonin were available for all six time points, total waking hours melatonin was estimated by calculating the sum of melatonin values, as well as by calculating area under the curve (AUC) for melatonin according to Pruessner, using AUC with respect to the ground [34]. Due to the skewed data distribution, melatonin values were grouped in quartiles for the generalized linear model analysis. Log values of melatonin were used in the longitudinal analysis.

### Correlation and generalized linear model analysis

Analyses were conducted on the whole population and in the exploratory analyses on the subgroup of patients with a current depressive episode. Spearman’s test (ρ) was used for pairwise correlations between hormone levels for each time point to total MADRS-S scores, age, bedtime, and in the post hoc analysis with the SDS subscale and global scores. A generalized linear model analysis for quartiles of melatonin in relation to baseline MADRS-S scores was also conducted, first without and then with the inclusion of possible confounding factors: gender, BMI, anti-depressive medication, use of oral contraceptives, and the influence of summer or winter season (April–September vs. October–March). For correlations between baseline hormone levels and MADRS-S scores at six months follow-up, a variation of follow-up time of ± two months was accepted. A logistic regression model was used to calculate the odds ratio for response, defined as ≥50% reduction in MADRS-S between baseline and follow-up. Baseline MADRS-S was then included in the model to control for the influence of baseline depressive symptoms. Log values of melatonin were used in these analyses. A two-sided P value of <0.05 was considered significant for all analyses.

## Results

### Hormone levels in saliva at different time points

**Fig 1:**
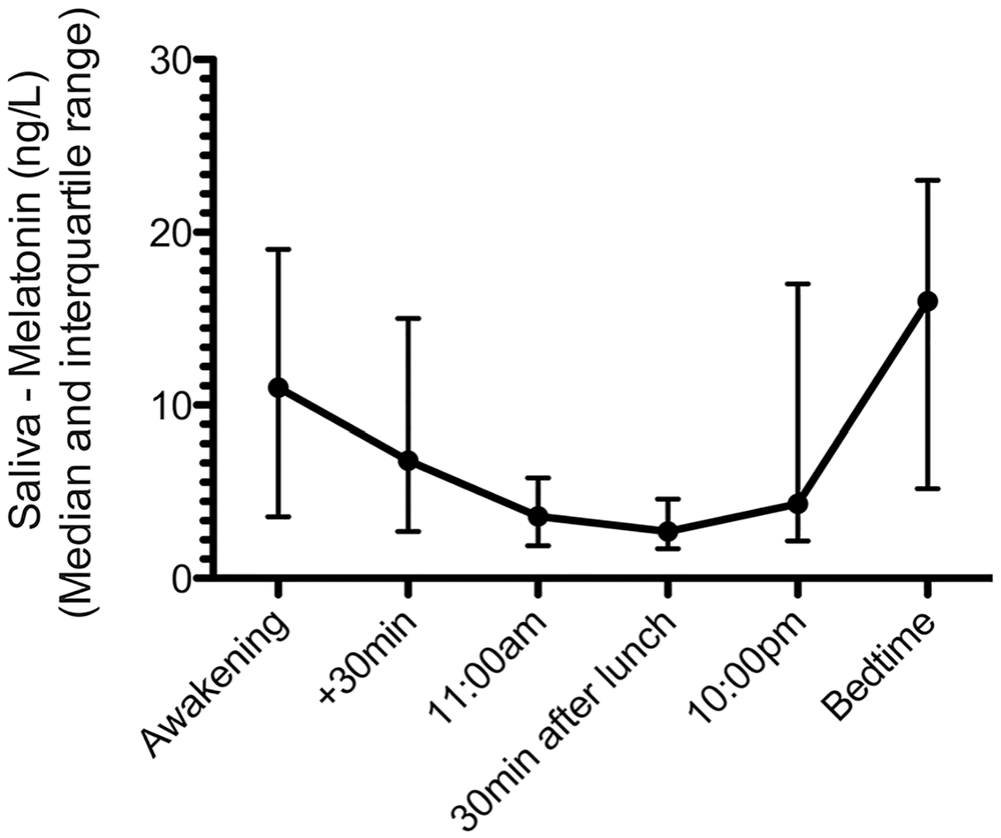
Median and interquartile range of melatonin levels in saliva for the different collection time points in patients (n = 119).

See Fig 1 for range of salivary melatonin related to individual time points. Salivary melatonin levels ranged from 0.5 to >50 ng/L. Eleven participants had melatonin levels >50 ng/L. Limited saliva volume did not allow further dilutions. Calculations for these participants were conducted using the value 50 ng/L. Age ranged from 18–25 years. No correlation between age and melatonin was found (ρ = -0.06, p = 0.51).

### Melatonin levels and depressive symptoms

**Fig 2:**
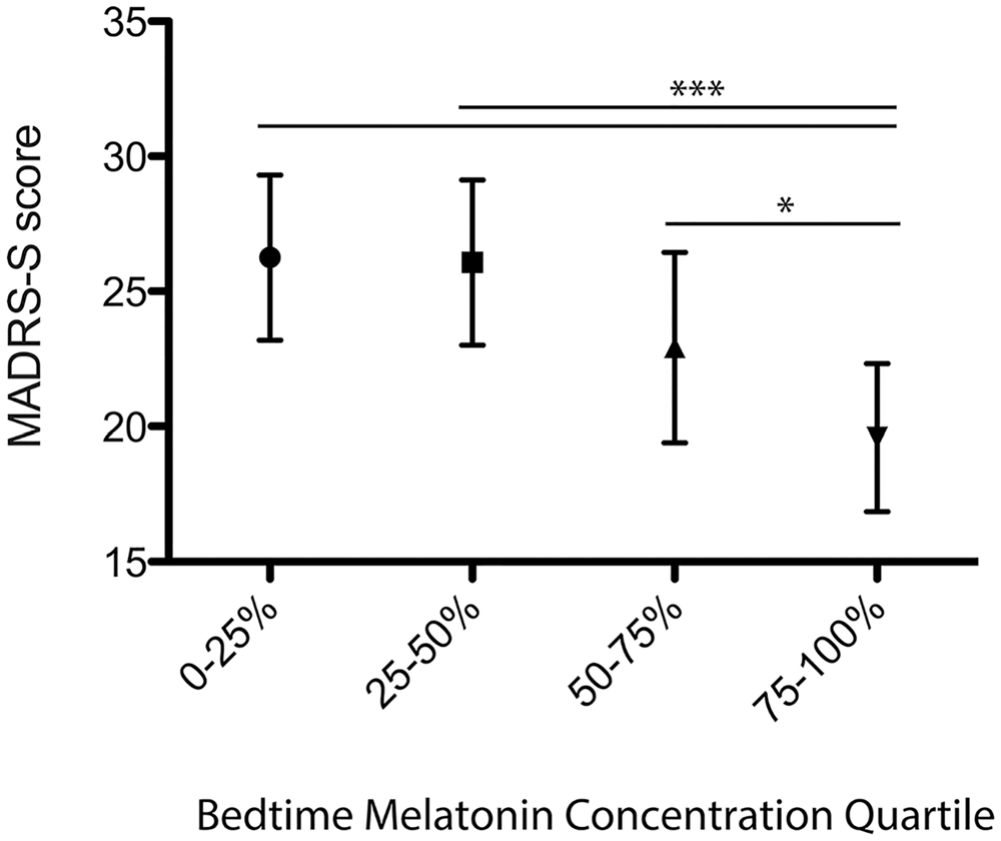
Bedtime-melatonin quartiles in patients show a negative relationship to MADRS-S total score at baseline. Whiskers indicate 95% confidence interval. Significant differences are indicated with lines (*p<0.05, *** p<0.001).

Fig 2 illustrates bedtime melatonin quartiles, which showed a negative dose response relationship to MADRS-S total score (p<0.001). The MADRS-S items were highly correlated; Cronbach’s Alpha was 0.852 and ranged from 0.82 to 0.855 if one item was deleted. No correlations with exact bedtimes were found. Most patients were found to have a melatonin peak in the evening, but different patterns of secretion were noted; melatonin peak in the morning, high melatonin throughout the day, or low melatonin at all time points. We hypothesized that low melatonin during waking hours would show larger association to symptoms than bedtime levels alone. Total production of melatonin during waking hours, calculated as AUC, approached but did not reach significance when correlated to total MADRS-S (ρ = -0.18, p = 0.07). However, the sum of melatonin levels at the six time points, another indication of production during waking hours, was negatively correlated to total MADRS-S scores (ρ = -0.22, p<0.05). Salivary melatonin levels at other individual time points showed no correlation to depressive symptoms.

Table 2 shows in a generalized linear model that patients with bedtime melatonin levels in the two lowest quartiles were at higher risk for an elevated MADRS-S score when compared to patients within the highest melatonin quartile (p<0.01). In a second model, the elevated risk remained after controlling for BMI, gender, use of SSRI/SNRIs, use of oral contraceptives, and season (p<0.01). Additionally, a smaller but significantly elevated risk for MADRS-S score for the third melatonin quartile in comparison to the fourth emerged (p<0.05).

**Table 2:** Generalized linear model for total MADRS-S score and salivary melatonin levels at bedtime in patients (Model 1). The relationship between low melatonin and high MADRS-S scores remains after including variables known as possible confounders (Model 2). First quartile denotes the quartile with lowest melatonin levels. Fourth quartile, with highest melatonin levels, is set as the reference. Season means summer vs. winter season.

		Model 1	Model 2
	Variables	OR 95% CI	OR 95% CI
MADRS-S score	Bedtime melatonin—1st quartile compared to 4th	1.37** (1.13–1.67)	1.39** (1.15–1.69)
	Bedtime melatonin—2nd quartile compared to 4th	1.36** (1.36–1.65)	1.36** (1.13–1.63)
	Bedtime melatonin—3rd quartile compared to 4th	1.20 (0.98–1.47)	1.25* (1.03–1.52)
	BMI		1.01 (1.00–1.02)
	Male gender		1.21 (0.1–1.46)
	Antidepressant medication		0.94 (0.82–1.09)
	Oral contraceptive use		0.87 (0.74–1.02)
	Season		0.99 (0.86–1.13)

* p<0.05;

** p<0.01

### Melatonin levels and disability

The SDS global disability score correlated negatively to bedtime melatonin (ρ = -0.27, p<0.01). Specifically, correlations were significant for self-rated disability for work/school (ρ = -0.25, p = 0.01) and in the family/home (ρ = -0.22, p = 0.02) but not in social function (ρ = -0.18, p = 0.06).

### Post hoc analysis of melatonin in subgroups of patients with DSM-IV diagnosis

Sixty-eight patients received a diagnosis of current depressive episode (n = 68) and were compared to patients without (n = 51). The median and range for total MADRS-S score differed significantly between the groups (28 [5–46] vs. 17 [7–34]; z = -5.87 p<0.001), while melatonin levels did not (13 [0.5–50] vs. 17 [0.5–50] ng/L; z = -1.82 p = 0.13). No differences were found between depressed and non-depressed patients in median bedtimes (23:11 and 23:06, respectively). Negative correlations between total MADRS-S to bedtime melatonin levels did not reach significance in subgroups depressed and non-depressed patients (ρ = -0.19, p = 0.15. and ρ = -0.22 p = 0.14 respectively).

Nighttime melatonin levels have been shown to be lower in the bipolar group [35, 36]. Of those with a current depressive episode, 16 patients were diagnosed with bipolar depression. Lower evening melatonin levels were seen in the group with bipolar depressive episode (n = 16) when compared to the group with unipolar depressive episode (n = 52) and to the non-depressed group (n = 51), but this difference was non-significant (p = 0.11).

### Post hoc analysis of baseline melatonin levels in relation to longitudinal data

Baseline melatonin values and MADRS-S data at follow-up was available for 27/68 = 40% of the patients with current depressive episode; 22 patients had unipolar depression while 5 had bipolar depression. Baseline bedtime melatonin showed a non-significant negative correlation to MADRS-S total score at follow-up (ρ = -0.37, p = 0.06). There was a positive correlation between baseline melatonin and the reduction in MADRS-S scores between baseline and at follow-up (ρ = 0.39. p<0.05). That is, higher bedtime melatonin levels were related to a larger reduction in total MADRS-S scores. The odds ratio for response was 4.4 (95% CI 1.06–18.43, p<0.05). In other words, with each log increase in bedtime melatonin, the likelihood for reduction of MADRS-S with at least 50% between baseline and follow-up increased 4.4 times. When controlling for baseline MADRS-S, the odds ratio was 4.3 (95% CI 0.98–18.97, p = 0.05).

### Post hoc analysis of blood samples for HbA1C and oxi-LDL

Blood samples were available for the first 89 individuals chronologically included in the study. Median levels of oxi-LDL level were 50.2 ng/ml (range: 24.1–226.5 ng/ml). Median HbA1C was 32.5 mmol/mol (range: 28–67 mmol/mol). The correlation between oxi-LDL values and melatonin values at both 10:00 p.m. and bedtime did not reach significance (ρ = -0.11, p = 0.29 and ρ = -0.14, p = 0.20, respectively). The correlation between HbA1C and melatonin values at both 10:00 p.m. and bedtime did not reach significance either (ρ = 0.15, p = 0.16 and ρ = 0.150, p = 0.15, respectively).

## Discussion

This observational study showed a negative correlation between evening melatonin levels and dimensional measures of depressive symptoms in young adult patients seeking psychiatric care. This relationship was independent of a diagnosis of current depressive episode. Importantly, these findings distinguish individuals within the patient population with otherwise similar psychiatric symptom presentation. In the post hoc longitudinal analysis, patients with low bedtime melatonin at baseline were less likely to show improvement in MADRS-S score at follow-up. These results are in line with the earlier studies that found a correlation between melatonin levels and depression severity in patients [5, 13, 16] and the results corroborate the proposed relationship between evening melatonin levels and depressive symptoms [2].

Melatonin is derived from tryptophan via serotonin. Activation of indoleamine 2.3-dioxygenase (IDO) and tryptophan dioxygenase (TDO) lead to increased kynurenines and potentially reduce levels of melatonin. Many risk factors for depression, such as stress, inflammation, and low levels of exercise, may activate IDO/TDO [37]. In line with our finding that low levels of melatonin are not specific to the diagnosis of current depressive episode but are instead true for a subgroup of patients, elevated activity in the kynurenine pathway is not seen for all patients with depression [38].

MADRS-S items were highly correlated. Interestingly, exclusion of individual MADRS-S items from the model in Table 2 did not significantly influence the results (data not shown). These symptoms are enriched in but not specific to depression, and may have a common denominator in cognitive dysfunction and loss of motivation and reward. Importantly, low bedtime melatonin was also related to higher global disability, especially at work or in school.

Other sources of melatonin, such as the gastrointestinal tract and pancreas, may also contribute to the total levels measured in saliva [39]. Patients with psychiatric disease have a higher risk of metabolic disease and early death [40]. Melatonin has recently been shown to have a wide spectrum of regulatory metabolic functions and is a powerful antioxidant [41]. Dysregulation of melatonin secretion may contribute to the higher risk of metabolic disease and early death seen in patients with psychiatric disease [40]. One possible consequence of reduced melatonin secretion in patients with severe symptoms is elevated oxidative stress. Melatonin can also influence transcription factors involved in insulin secretion in the pancreas in a receptor-dependent manner [41–43]. There seems to be a link between melatonin and glucose homeostasis [44]. One example of this is that a variation in the melatonin receptor 1B gene has been associated with levels of HbA1C [45]. Recently, we described mRNA and protein expression of melatonin receptors MT_2_ and MT_1_ in the human pancreas [39]. Genetic variants of MT_2_ are reported to increase the risk of developing type 2 diabetes. More receptors on the cell surface increase the sensitivity for melatonin, which reduces the capacity to release insulin [46, 47]. In this young adult population, the majority of patients had both oxi-LDL and HbA1C levels within the normal range. An older population or a follow-up study on this population in the future is needed to determine the long-term consequences of melatonin levels on metabolic status in patients with severe states. This study is of extra importance as it concerns young adults, and the identification of early prognostic markers may have a large impact on future health.

Current diagnostic categories poorly identify underlying biological processes. With the goal to bring more precision into psychiatric research, the National Institute of Mental Health (NIMH) has created the Research Domain Criteria (RDoC). The study followed RDoC principles by using dimensional measures and an “agnostic” treatment of current diagnostic categories. In RDoC, these symptoms may reflect loss of function in the positive valence systems. The data was collected prospectively, and diagnostic methods were standardized. The population was regarded as representative as the gender, age, and diagnosis were similar to those in a previous study from the same catchment area and population, with a participation rate of 92% [48]. Furthermore, we have been able to control for known possible confounding factors including gender, BMI, SSRI/SNRI treatment, use of oral contraceptives, season, bedtime, and patients who are of the same narrow age span. This study also indicates that melatonin levels in saliva can be efficiently and reliably studied using saliva sampling at home in an outpatient setting.

Patients may have completed saliva testing differently at home. Extensive measures were taken to reduce this risk. Strong correlations between both time points in the morning and evening, respectively, provide internal validity for the hormone measures. Less than five percent of hormone values were missing. Limitations include low inclusion of patients from the possible participants (119/722). It can be speculated that this is an age group (18–25) where compliance to bringing back samples may be lower than in other age groups.

One interpretation of earlier studies is that hypersecretory states may exist. Notably, a study of healthy Japanese students found a weak positive correlation between salivary melatonin and self-assessment of state and trait anxiety and sub-clinical symptoms of depression [49]. The ability to detect hypersecretion in our study was limited by the method in two ways: the detection limit was 50 ng/L due to the limited amount of saliva available and samples were gathered at bedtime, whereas melatonin amplitude is generally maximal during sleep.

Low bedtime melatonin in saliva could also represent a phase delay in depressed patients, which has been shown earlier [8]. However, both phase advance [15] and no significant phase shift of melatonin in depressed patients compared to controls [3, 5] has been reported. The method for saliva assessment limited the possibility of addressing this question.

Analysis of MADRS-S at six months post baseline was not postulated before starting the study, but the method and hypothesis were defined before studying this outcome. A wide time span (4–8 months) was defined as follow-up. Only patients with depression were available, as MADRS-S is used in the clinical monitoring of only those with current depressive episode. Therefore, the prognostic value of bedtime melatonin in the whole population is not known.

## Conclusion

The present study corroborates a negative correlation between low bedtime melatonin levels and the severity of depressive symptoms in a young adult general psychiatric population. These associations remain after adjustment for potential confounders. Post hoc analysis indicates prognostic implications. This study also shows that melatonin levels in saliva can be efficiently and reliably studied using saliva sampling at home in an outpatient setting. New medications that target melatonin receptors are now available, and an interesting hypothesis is that patients with depressive symptoms and lower levels of melatonin may benefit most from these treatments. With further research, salivary melatonin could become a clinically useful biomarker.
